# Identification of a new vector species of avian haemoproteids, with a description of methodology for the determination of natural vectors of haemosporidian parasites

**DOI:** 10.1186/s13071-019-3559-8

**Published:** 2019-06-18

**Authors:** Rasa Bernotienė, Rita Žiegytė, Gabrielė Vaitkutė, Gediminas Valkiūnas

**Affiliations:** Nature Research Centre, Akademijos 2, 08412 Vilnius, Lithuania

**Keywords:** Vectors, *Haemoproteus*, *Culicoides*, Sporozoites, PCR

## Abstract

**Background:**

Haemosporidian parasites are transmitted by dipteran blood-sucking insects but certain vectors remain unidentified for the great majority of described species. Sensitive PCR-based methods are often used for the detection of haemosporidian infection in wild-caught insects. However, this approach alone cannot distinguish between different sporogonic stages and thus is insufficient to demonstrate that the parasites produce the infective stage (sporozoite), which is essential for transmission. To prove that PCR-positive insects could act as vectors, the record of sporozoites is needed. We developed a methodology for the determination of natural vectors of avian *Haemoproteus* species and other haemosporidians. The essence of this approach is to apply PCR-based and microscopic diagnostic tools in parallel for sporozoite detection in insects.

**Methods:**

*Culicoides* biting midges transmit avian *Haemoproteus* parasites, but certain insect species, which are involved in transmission, remain insufficiently investigated. Biting midges were collected in the wild and identified; parous females were dissected and preparations of thorax content containing salivary glands were prepared. Remnants of the dissected midges were screened using PCR-based methods. Only thorax preparations of PCR-positive biting midges were examined microscopically.

**Results:**

In total, 460 parous females belonging to 15 species were collected and dissected. DNA of haemosporidians was detected in 32 (7%) of dissected insects belonging to 7 species. Of the thorax samples PCR-positive for *Haemoproteus* parasites, two preparations were microscopically positive for sporozoites. Both biting midges were *Culicoides kibunensis. Haemoproteus pallidus* (hPFC1) was identified, indicating that transmission of this infection occurs at the study site. It was proved that seven species of biting midges take bird blood meals naturally in the wild.

**Conclusions:**

*Culicoides kibunensis* is a new vector species of avian haemoproteids and is a natural vector of *H. pallidus*. Numerous studies have identified vectors of *Haemoproteus* parasites experimentally; however, this is the first direct identification of a natural vector of *Haemoproteus* infection in the Old World. We suggest using the described methodology for vector research of *Haemoproteus* and other haemosporidians in the wild.

**Electronic supplementary material:**

The online version of this article (10.1186/s13071-019-3559-8) contains supplementary material, which is available to authorized users.

## Background

Haemosporidian parasites (Haemosporida) are obligate heteroxenous protists that inhabit all major groups of terrestrial vertebrates and use blood-sucking dipteran insects as vectors. Avian haemosporidians are the largest group in the order Haemosporida by number of described species [1]. Over 200 species of avian haemosporidians have been described and classified into four genera: *Plasmodium*, *Haemoproteus*, *Leucocytozoon* and *Fallisia* [2, 3]. Some *Leucocytozoon* parasites are highly pathogenic in poultry [3, 4]. *Plasmodium* and some *Haemoproteus* species have been reported to cause pathology and even lethal diseases in non-adapted avian hosts [5–7]. Information about the pathogenicity of *Fallisia* infections is absent. Representatives of different genera of avian haemosporidians use different groups of dipteran insects for transmission: avian *Plasmodium* parasites are transmitted by mosquitoes (Culicidae) [2, 8], *Haemoproteus* spp. by louse flies (Hippoboscidae) and biting midges (*Culicoides*, Ceratopogonidae) [9, 10], and *Leucocytozoon* species mainly by black flies (Simuliidae) [11, 12]. Haemosporidian sporogonic development remains the most poorly investigated part of the life-cycle in these pathogens [13]. The majority of current studies focus on the interaction between haemosporidians and vertebrate hosts, but vector species and the host-parasite interaction during sporogonic development remains insufficiently investigated, particularly in wildlife [14].

*Culicoides* biting midges (Diptera: Ceratopogonidae), vectors of *Haemoproteus* parasites of the subgenus *Parahaemoproteus*, are the smallest blood-sucking flies [15]. They transmit arboviruses, bacteria, protozoans and helminth parasites of humans and animals [16], but due to their tiny size and fragility, remain the least studied among the major dipteran vector groups [16]. *Culicoides* biting midges are widespread and diverse, with more than 1400 species described and at least 58 species present in Europe [17]. Only one Old World native *Culicoides* species (*Culicoides nubeculosus*) is known to be colonised in laboratory [18]. This biting midge species as well as *Culicoides impunctatus*, one of the most abundant species of *Culicoides* in the North Europe, are excellent experimental vectors and likely are natural vectors of several *Haemoproteus* parasites in Europe [19–21]. A limited amount of information about *Culicoides* biting midges as vectors of *Haemoproteus* parasites is available due to former experimental studies performed with *Culicoides arboricola*, *C. crepuscularis*, *C. downesi*, *C. edeny*, *C. haematopodus*, *C. hinmani*, *C. knowltoni*, *C. stilobezziodes* and *C. sphagnumensis* in the New World [3, 5, 21–24]. However, natural vectors of haemosporidian parasites remain insufficiently investigated worldwide [23].

Experimental studies on the transmission of *Haemoproteus* species by biting midges are difficult to design, particularly because of difficulties to collect, infect experimentally, maintain in the laboratory of wild-caught individuals as well as delicate procedures of their dissection and microscopic preparation due to the tiny size and fragility of the midges [20, 21]. The great majority of current studies about *Culicoides* spp. as possible vectors of *Haemoproteus* parasites are based only on molecular determination of the parasite lineages reported in wild-caught insects. According to PCR-based testing, seven *Culicoides* species have been reported to harbour *Haemoproteus* parasite DNA in Europe: *Culicoides alazanicus* [25]; *Culicoides circumscriptus* [14, 25, 26]; *C. festivipennis* [25, 26]; *C. kibunensis* [27, 28]; *C. pictipennis* [26, 28]; *C. segnis* [27]; and *C. scoticus* [28]. Reports of parasite DNA indicate that the PCR-positive insects might be possible vectors; however, these data alone are insufficient to prove the insects could transmit haemosporidian infections. The main problem is that the currently used PCR-based diagnostics cannot distinguish between different stages of sporogonic development. Specifically, it cannot distinguish between invasive for vertebrate hosts (sporozoites) and non-invasive (gametocytes, gametes, ookinetes and oocysts) sporogonic stages. In other words, PCR-based diagnostics is insufficient to demonstrate that the parasites are capable of reaching the stages necessary for transmission [29]. Recent experimental studies indicate that avian malaria parasites (*Plasmodium* spp. and related haemosporidians belonging to *Haemoproteus*) can persist even in resistant blood-sucking insects for several weeks after initial blood meals due to the survival of ookinetes. DNA of these parasites can be readily detected by PCR-based diagnostics [13, 14, 29, 30], but this does not necessarily mean that the PCR-positive insects act as vectors. The detection of haemosporidian DNA in blood-sucking dipterans can be used as a molecular tag in determining bird-biting insects as well as to identify probable links between these insects and parasites, but not as proof that an insect acts as a haemosporidian vector [17, 31].

Knowledge about natural vectors of haemosporidians is essential for a better understanding of the patterns of epidemiology of vector-borne diseases and the evolution of vector-parasite interactions [13, 23], but presently remains insufficient, particularly in the wild. This is unfortunate because various haemosporidiosis are important for the health of domestic and wild animals [4–7]. During the present study, we developed and tested a simple methodology for the detection of the invasive haemosporidian vector stage (sporozoites) of *Haemoproteus* parasites in wild-caught biting midges. The essence of this approach was to apply both PCR-based methods and microscopic examination in parallel during vector research. The main aim was to determine which biting midges harbour sporozoites and are likely natural vectors for avian *Haemoproteus* parasites. This methodology involved four steps: (i) collection of insects in the wild; (ii) dissection of parous *Culicoides* females and preparation of their thorax content; (iii) determination and identification of haemosporidian parasites in individual insects using PCR-based methods; and (iv) microscopic examination of the thorax preparations of PCR-positive individuals. To our knowledge, this study is the first direct identification of natural vectors of haemosporidian parasites in wildlife combining PCR-based and microscopic tools.

## Methods

### Material collection

Biting midges were collected using one black light trap in Vilnius, Verkiai Regional Park (54°45′N, 25°17′E), Lithuania between 12 May and 30 October in 2016. Insects were caught one night every week during this period of time. The trap was turned on 1–2 h before sunset and was turned off 2–3 h after sunrise. Biting midges were collected into a pot containing water supplemented with a drop of liquid soap. Samples were transported to the laboratory the same morning as collection. Fresh material was investigated under a binocular stereoscopic microscope (SZX10; Olympus, Tokyo, Japan). All parous females were dissected forthwith except for those collected on 26th May, 2nd June and 22nd June, when the abundance of biting midges was extremely high and each sample contained hundreds of parous females; for each of these three days we dissected between 51 and 55 fresh parous biting midges. Parous females were recognised due to the presence of the readily visible burgundy pigment in the subcutaneous cells of the abdomen, indicating a digested blood meal prior to capture [32]. Parous biting midges search for a new blood meal after the first gonotrophic cycle is completed and therefore they are more likely to yield sporozoites of haemosporidian parasites.

### Dissection of insects

Individual parous females were identified on the basis of their wing and head patterns and other morphological features [33, 34]. Below, the dissection procedure is described. Each insect was placed in a drop of 0.9% normal saline. The head and wings were removed and stored in 96% alcohol for insect identification. The salivary glands are located in the anterior/upper part of the thorax [3], so a portion of the content from this part of the thorax was removed and gently crushed using dissecting needles to prepare a small smear [20]. Salivary glands of biting midges are tiny, and they were not always readily visible among adjacent thorax tissues, which were removed together with the glands during the dissection process. It is important that the smears are thin for microscopic examination. In order to avoid contamination, all dissecting needles were disinfected using fire after each dissection. Thorax preparations were air dried, fixed with absolute methanol and stained with Giemsa stain [3, 20]. Remnants of dissected biting midges were stored in 96% alcohol for PCR-based analysis. This analysis is sensitive enough for detecting parasite DNA from dissected insects because some sporozoites likely remain in the crushed thorax [19–21].

### DNA extraction, PCR and sequencing

Total DNA was extracted from thoraxes using the ammonium acetate extraction method [35]. The extracted DNA was then dissolved in 20 μl of TE solution. For genetic analysis, we used a nested PCR protocol amplifying a fragment of the cytochrome *b* gene of the *Haemoproteus* and *Plasmodium* parasites [36, 37]. For detection of possible co-infections in biting midges a nested-multiplex PCR was applied [38] since it can detect the presence of *Haemoproteus* and *Plasmodium* co-infections in the same sample. All samples were evaluated by gel electrophoresis using 3 μl of PCR product in a 2% agarose gel. One negative control (nuclease-free water) and one positive control (an infected sample, which was *Haemoproteus tartakovskyi*-positive by microscopic examination of blood films) were used per every 14 samples. No cases of false positive or negative samples were reported.

To confirm the identity of biting midges, we used the insect-specific primers LCO149 and HCO2198, which amplify a fragment of cytochrome *c* oxidase subunit 1 of mitochondrial DNA [39]. Morphological identification was consistent with the PCR-based identification of the insects.

DNA fragments of all PCR-positive samples were sequenced. Sequences were edited and aligned using BioEdit [40] and deposited in the GenBank database under the accession numbers KM116567-KM116579). The genetic analyzer “Basic Local Alignment Search Tool” (National Centre of Biotechnology Information, http://www.ncbi.nlm.nih.gov/BLAST) was used to determine lineages of detected DNA sequences.

### Microscopic examination of thorax preparations

Thorax preparations of insects that were PCR-positive for *Haemoproteus* parasite DNA were examined using an Olympus BX-43 light microscope equipped with an Olympus SZX2-FOF digital camera and imaging software Image-Pro plus 7.0 (Media Cybernetics). Smears were examined at high magnification (1000×). Representative preparations of sporozoites (accession nos. 49025-49026NS) were deposited at the Nature Research Centre, Vilnius, Lithuania. Morphometric parameters of sporozoites were compared using a t-test for independent samples. Prevalence of infections in midges during the season were compared by Yates corrected Chi-square test. A *P-*value of < 0.05 was considered significant.

## Results

In all, 460 parous *Culicoides* females belonging to 15 species were collected and dissected (Table 1, Additional file 1: Table S1). The most abundant species were *Culicoides pictipennis* (41.3% of all *Culicoides* individuals dissected in May), *Culicoides kibunensis* (30.8% of all *Culicoides* insects dissected in June), *Culicoides obsoletus* and *Culicoides scoticus.* The latter two species represented between 57.1 % and 95.8% of all collected and dissected parous biting midges between August and October.

**Table 1:** Parous *Culicoides* biting midges dissected in Verkiai Regional Park, 2016

Species	*n*
*C. albicans*	2
*C. chiopterus*	23
*C. circumscriptus*	5
*C. fascipennis*	2
*C. festivipennis*	20
*C. impunctatus*	36
*C. kibunensis*	68
*C. newsteadi*	1
*C. obsoletus*	123
*C. pallidicornis*	2
*C. pictipennis*	28
*C. punctatus*	67
*C. reconditus*	3
*C. scoticus*	63
*C. segnis*	17
Total (15 species)	460

*Abbreviation*: n, number of biting midges

**Fig. 1:**
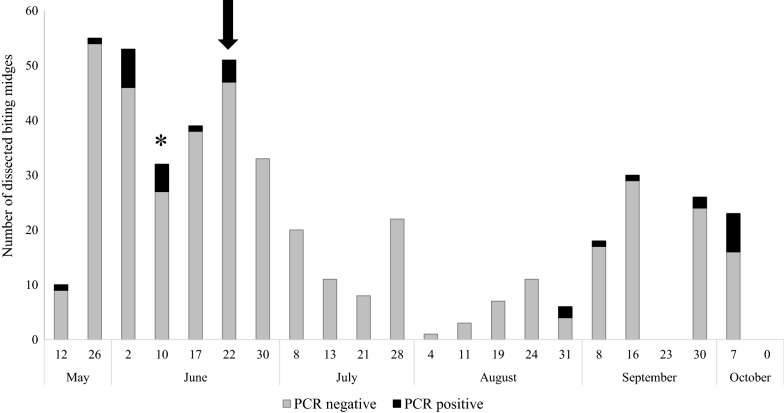
Number of collected and dissected biting midges during the season. All PCR-positive insects were examined microscopically. Arrow shows the time when sporozoites were reported in *C. kibunensis* biting midges; asterisk indicates the time when two co-infections of *Plasmodium* and *Haemoproteus* parasites were detected in the same biting midges

The highest abundance of *Culicoides* midges was reported from the end of May until the end of June, and that coincided with the highest prevalence of infection with avian haemosporidian parasites in biting midges (Fig. 1). The parasites were not reported from the end of June until the end of August; the prevalence increased again at the beginning of October (Yates corrected Chi-square, *χ*^2^ = 2.83, *df* = 1, *P* < 0.01). No parous biting midges were collected after the 7th October.

DNA of haemosporidian parasites was detected in 32 biting midges. *Plasmodium* parasites were detected in 5 biting midges and *Haemoproteus* parasites were reported in 27 biting midges using nested PCR (Table 2). Eleven genetic lineages of *Haemoproteus* and 4 of *Plasmodium* parasites were detected in 7 *Culicoides* species showing that these insects had taken infected blood meals because that is the only way that they can acquire bird blood parasites.

**Table 2:** Avian haemosporidian parasites and their lineages detected in biting midges using PCR-based diagnostics

*Haemoproteus* species		*Culicoides* species						
Species	Lineage	*C. festivipennis*	*C. impunctatus*	*C. kibunensis*	*C. obsoletus*	*C. pictipennis*	*C. punctatus*	*C. scoticus*
*H. attenuatus*	hROBIN1	1^a^			1			
*H. balmorali*	hLULU1							1
*H. lanii*	hRB1				3			
*H. majoris*	hWW2						1	
*H. minutus*	hTUPHI01					1		
	hTURDUS2			2	2		1	3
*H. pallidus*	hPFC1			2			1^a^	
*H. parabelopolskyi*	hSYAT01					1		
*H. tartakovskyi*	hHAWF1						1	1
	hSISKIN1			1	1			2
*Haemoproteus* sp.	hSFC4				1			
*P. circumflexum*	pTURDUS1							1
*P. matutinum*	pLINN1		1					
*P. relictum*	pGRW11				1			1
	pSGS1							1

^a^Co-infection with *Plasmodium* parasites detected using nested-multiplex PCR

Additionally, the nested-multiplex PCR, showed the presence of co-infection of *Plasmodium* and *Haemoproteus* parasites in two biting midges collected during the second week of June (Table 2, Fig. 1); only *Haemoproteus* genetic lineages hPFC1 and hROBIN1 were detected in these biting midges using nested PCR.

A high prevalence of haemosporidian parasites was determined in May (10%). *Culicoides pictipennis* biting midges were infected during this time. The prevalence of haemosporidians was also high during the first and the second weeks of June (13.2% and 15.6%, respectively). Four *Culicoides* species were determined to be infected during this time: *C. punctatus*; *C. obsoletus*; *C. festivipennis*; and *C. impunctatus.* The prevalence of haemosporidians in insects collected on 22nd June was 7.8% and only *C. kibunensis* biting midges (4 insects) were infected. *Culicoides punctatus*, *C. obsoletus* and *C. scoticus* biting midges were found to be infected with haemosporidians from the end of August until October, with an infection prevalence up to 33.3%.

**Fig. 2:**
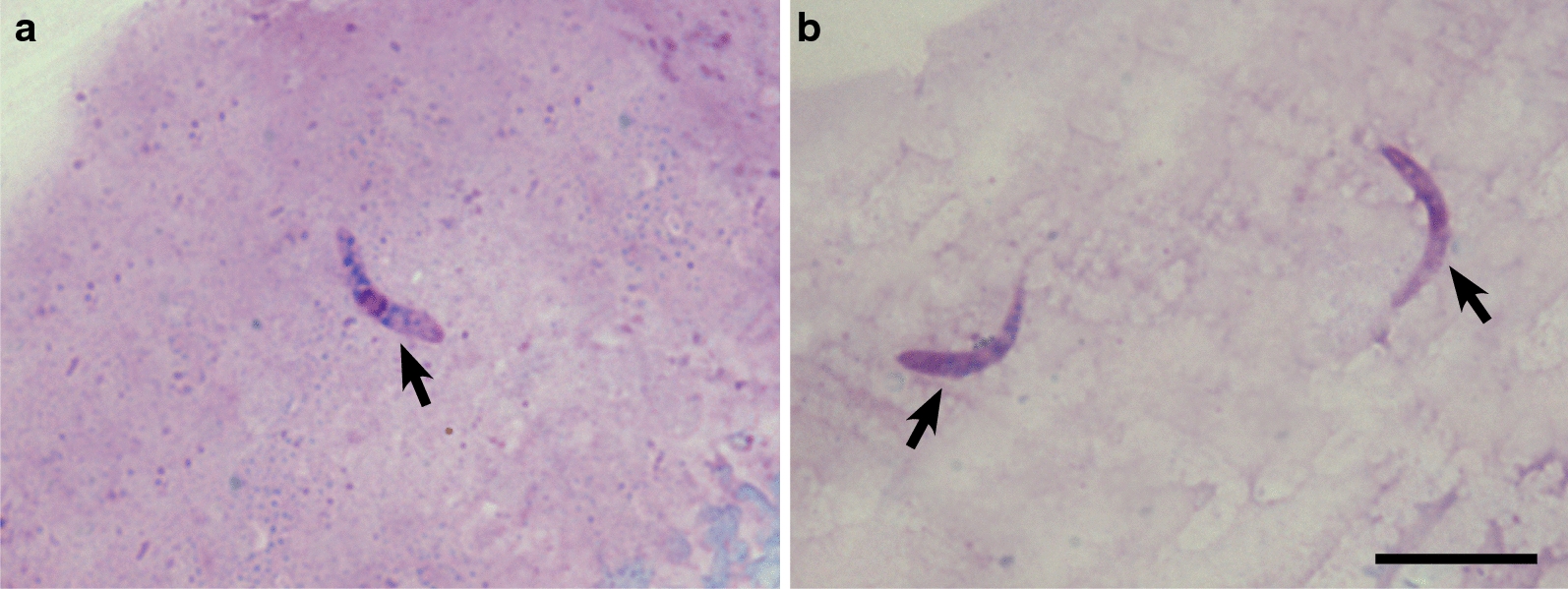
Sporozoites of *Haemoproteus pallidus* (hPFC1) in thorax preparations of two different individuals of *Culicoides kibunensis*. Parasites reported in each individual of *Culicoides* are shown in panels **a** and **b**. Note the similar size and morphology of sporozoites reported in different insects. Arrows indicate nuclei of the parasites. *Scale-bar*: 10 µm

*Haemoproteus* sporozoites were detected in two of 27 PCR-positive insects (Fig. 2). Both biting midges were collected on 22nd June. These were identified as *Culicoides kibunensis* using morphological features and barcoding sequences. Nested PCR results showed the presence of the same parasite genetic lineage in both insects: *Haemoproteus pallidus* (lineage hPFC1). Sporozoites (*n* = 21) looked like thin elongate bodies, ranging between 8.5–11.7 (mean ± SD, 10.3 ± 1.1) µm in length and between 1.0–1.7 (1.3 ± 0.2) µm in width, with nuclei located slightly off-centre (Fig. 2, Additional file 2: Table S2).

## Discussion

The methodology described provides opportunities to identify natural vectors of haemosporidians in the wild. In spite of being relatively time-consuming, this is currently the most straightforward approach to gain such information in the wild. It is relatively easy to master this methodology, and we recommend its use in haemosporidian vector research.

*Culicoides kibunensis* is a new vector of avian haemoproteids. This biting midge has been shown to harbour *Haemoproteus* spp. DNA in Czech Republic [27] and Germany [28]. Both studies used only PCR-based methods during detection of the parasites, and the presence of sporozoites was not documented. DNA of *Haemoproteus minutus* was detected in this biting midge in Germany (genetic lineage hTUPHI01) [28], Czech Republic (hTURDUS2) [27] and the present study (hTURDUS2). DNA of *Haemoproteus parabelopolskyi* (hSYAT01) was also detected in *C. kibunensis* in Germany [28], and DNA of *Haemoproteus* sp. (hCUKI1) was reported in Czech Republic [27]. Available data indicate that *C. kibunensis* is likely an important natural vector of *Haemoproteus* parasites in Europe and thus worthy of attention in epidemiological studies of haemoproteosis.

*Haemoproteus pallidus* (hPFC1) was detected in *C. kibunensis* biting midges for the first time. This infection is common in birds belonging to the Muscicapidae [41, 42]. Sporogony of *H. pallidus* parasites completes in *C. kibunensis*, with numerous sporozoites reported in thorax preparations (Fig. 2), indicating a possible high vectorial capacity. In spite of numerous reports of experimental vectors [21], this is the first identification of a natural vector of avian haemoproteids in the Old Word. It worth noting that sporozoites of the same parasite species also developed in experimentally infected *C. impunctatus* [20], and are morphologically indistinguishable in both of these insects by morphology and size (t-test: length, *t*_(40)_ = 1.65, *P* = 0.1; width, *t*_(40)_ = 1.82, *P* = 0.07).

DNA of 11 different *Haemoproteus* parasites was detected in biting midges belonging to six species during this study (Table 2). These biting midges are worthy of attention in *Haemoproteus* parasites vector research. It worth mentioning that *Culicoides festivipennis*, *C. scoticus* and *C. pictipennis* have been previously reported to harbour *Haemoproteus* lineages. DNA of haemoproteids belonging to genetic lineages hCIRCUM01, hCIRCUM03 and hTURDUS2 has been detected in *Culicoides festivipennis* in Bulgaria [25] and Czech Republic [27]. The lineage hCULSC001 has been detected in *C. scoticus* in Germany [28] and hTUPHI01 and hTURDUS2 have been detected in females of *C. pictipennis* in Germany and Bulgaria, respectively [26, 28]*. Haemoproteus minutus* lineage hTUPHI01 was also detected in *C. pictipennis* during this study. The diversity of parasites detected in *C. scoticus* biting midges was high: four genetic lineages of *Haemoproteus* were reported, suggesting that this insect might be an important vector of avian haemoproteosis.

Four genetic lineages of *Plasmodium* parasites were reported in biting midges during this study (Table 2). *Culicoides* biting midges do not transmit avian malaria parasites of *Plasmodium* [23], but have been reported to harbour the DNA of malaria parasites [13, 14, 30]. These are examples of the presence of *Plasmodium* infection in the wrong invertebrate hosts reported by PCR diagnostics and possible illustrations of abortive haemosporidian development in the wrong hosts [29]. These data further call for the parallel application of PCR-based and microscopic data to determine the natural vectors of haemosporidian parasites. Experimental observations show that non-degraded DNA of haemosporidians can be found in the head, thorax and abdomen of mosquitoes for over two weeks after taking an infected blood meal, due to the persistence of ookinetes, which actively move from the gut contents to haemocoel, resulting in the presence of parasites throughout the body of the insect [29]. Massive ookinete infection both in the abdomen and thorax of *Culicoides* biting midges has also been documented [43]. These data can explain the presence of haemosporidian DNA, including the parasites that cannot complete sporogony, in the thorax of the dissected insect. The low detectability of sporozoites in *Haemoproteus* spp. positive biting midges (Table 2) might be partly due to the amplification of DNA from non-invasive parasite stages. Microscopic analysis helps to determine invasive stages (sporozoites) in salivary glands and to distinguish the most probable competent vectors.

In spite of the fact that *C. circumscriptus* and *C. segnis* are known to harbour *Haemoproteus* parasite DNA in the wild [14, 27], we did not detect infections in females of these two species. This can be due to the small numbers of collected parous females (Tables 1, 2). This study added two species (*C. obsoletus* and *C. punctatus*) to the list of potential vectors of *Haemoproteus* parasites: five and four genetic lineages of *Haemoproteus* have been detected in these biting midges, respectively (Table 2). *Culicoides obsoletus* and *C. punctatus* are among the most abundant biting midges in the north of Europe [44, 45], so they can be important in *Haemoproteus* parasite transmission, thus worthy of attention in haemoproteosis epidemiology research.

The prevalence of haemosporidian infections is often high in many bird species worldwide, with the overall prevalence even reaching 80–100% in many bird populations during the breeding season in Europe [3, 46]. Due to the specificity of haemosporidian lineages to certain bird groups, reports of certain lineages in insects provide the opportunity to determine the origin of the blood meal. In other words, the molecular markers of avian haemosporidian parasites can be used for determining the feeding preference of biting midges to the species level of their avian hosts [17, 31]. It is important to note that this study contributes new information about natural host specificity of biting midges. Fifteen genetic lineages of avian haemosporidian parasites were detected in seven *Culicoides* species, showing that these species of biting midges had naturally fed on bird blood. *Culicoides festivipennis*, *C. impunctatus*, *C. kibunensis*, *C. obsoletus*, *C. pictipennis*, *C. punctatus* and *C. scoticus* fed on bird blood at our study site. The PCR-based prevalence of haemosporidians in five *Culicoides* species was relatively high (7.4% in *C. kibunensis*, 7.3% in *C. obsoletus*, 7.1% in *C. pictipennis*, 6% in *C. punctatus* and 15.9% in *C. scoticus*) showing that ornithophily of these biting midges is a pattern. *Culicoides kibunensis* and *C. pictipennis* biting midges have been reported to preferably feed on birds [13, 47], but only sporadic cases of ornithophily of biting midges belonging to other mentioned species have been reported [44, 47]. Currently, *C. obsoletus*, *C. punctatus* and *C. scoticus* are believed to feed on mammals [13, 44, 47, 48]. However, this study showed that more than 14% of collected parous *C. scoticus* biting midges were infected with avian haemosporidians indicating that this midge species can successfully feed on birds in the wild.

Many haemoproteid species and their lineages are relatively specific, and they complete their life-cycle and produce gametocytes (the only infective stage for vectors) in particular avian hosts (Table 3). In other words, if insects are PCR-positive with a particular parasite lineage, they likely took blood meals on a particular bird species, because the blood meal is the only pathway for vectors to acquire avian haemosporidian infections [21, 31]. This therefore provides opportunities to obtain detailed information about the feeding preference of blood-sucking insects on specific taxonomic bird groups due to the detection of *Haemoproteus* parasite lineages in these insects. For example, the lineage hSISKIN1 of *Haemoproteus tartakovskyi* has been reported to complete development and produce gametocytes only in siskins (*Carduelis spinus*) and crossbills (*Loxia curvirostra*) [29, 49–51]. This parasite was detected in *C. kibunensis*, *C. obsoletus* and *C. scoticus* biting midges showing that these biting midges likely fed on blood-meals obtained on siskins and crossbills (Table 3). Information about the feeding preference of biting midges sampled during this study is summarised in Table 3. The host range of biting midge species is difficult to determine and remains insufficiently investigated. Additional data providing information about the host preference of *Culicoides* biting midges (Table 3) are important for epidemiology studies. Recent findings highlight that the majority of *Culicoides* species are able to feed on several vertebrate host species but show preferences either for mammals or birds [17, 52]. Host generalist biting midges are of particular interest in epidemiology research because they can feed on different vertebrates [28], thus could transmit agents of diseases between different taxonomic groups of vertebrates and facilitate the spread of emerging diseases. Avian *Haemoproteus* parasites and their lineages are convenient biological tags for better understanding the feeding preferences of *Culicoides* species in the wild [31] (Table 3).

**Table 3:** Occurrence of *Haemoproteus* parasites and their cytochrome *b* lineages in European *Culicoides* species using PCR-based diagnostics (this study) and in birds using both PCR-based diagnostics and microscopic examination (literature data). Reported biting midges were infected by parasites of corresponding lineages, which develop gametocytes only in birds of the corresponding genera

*Culicoides* spp.	*Haemoproteus* spp.	*Haemoproteus* spp. lineage	Bird genus^a^	References
*C. festivipennis*	*H. attenuatus*	hROBIN1	*Erithacus*, *Luscinia*	[21, 29]
*C. kibunensis*	*H. minutus*	hTURDUS2	*Turdus*	[21, 42, 49, 53, 54]
	*H. pallidus*	hPFC1	*Ficedula*	[41, 42, 49, 53]
	*H. tartakovskyi*	hSISKIN1	*Carduelis*, *Loxia*	[29, 42, 49–51, 54, 55]
*C. obsoletus*	*H. attenuatus*	hROBIN1	*Erithacus*, *Luscinia*	
	*H. lani*	hRB1	*Lanius*	[42, 50, 53]
	*H. minutus*	hTURDUS2	*Turdus*	
	*H. tartakovskyi*	hSISKIN1	*Carduelis*, *Loxia*	
*C. pictipennis*	*H. minutus*	hTUPHI01	*Turdus*	[54]
	*H. parabelopolskyi*	hSYAT01	*Sylvia*	[41]
*C. punctatus*	*H. majoris*	hWW2	*Acrocephalus*, *Parus*, *Phylloscopus*, *Sylvia*	[41]
	*H. minutus*	hTURDUS2	*Turdus*	
	*H. pallidus*	hPFC1	*Coccothraustes*	
	*H. tartakovskyi*	hHAWF1	*Coccothraustes*	[49]
*C. scoticus*	*H. balmorali*	hLULU1	*Luscinia*	[53]
	*H. minutus*	hTURDUS2	*Turdus*	
	*H. tartakovskyi*	hHAWF1	*Coccothraustes*	
	*H. tartakovskyi*	hSISKIN1	*Carduelis*, *Loxia*	

^a^Only genera whose species support complete development and show gametocytes of corresponding parasites in the blood are included

Seasonal variation in the prevalence of haemosporidian infections in birds has been well documented, with a marked increase in parasite prevalence during spring-beginning of summer in temperate regions of the Holarctic [3, 23, 56, 57]. However, few studies compared these seasonal changes in regard to the abundance of putative vectors and parasite prevalence in vectors [5, 58]. The present study shows that the highest prevalence of haemosporidian parasites in biting midges (Fig. 1) was observed in May (overall, up to 10% of midges were PCR-positive) and the beginning of June (up to 16%). This is the period of bird breeding when numerous juveniles appear at our study site. The presence of numerous naive birds and susceptible vectors facilitates infection transmission to juvenile birds, and this is important for the survival of disease agents. *Culicoides pictipennis*, *C. punctatus*, *C. obsoletus*, *C. festivipennis*, *C. impunctatus* and *C. kibunensis* were the dominant biting midges in May–June, and these insects are the most probable vectors during this time period.

It worth noting that the prevalence of *Haemoproteus* parasites in biting midges was relatively high at the end of August and September–October, with a particularly high prevalence during the first week of October (Fig. 1). These are periods of time when active seasonal migration takes place and numerous birds from the northern populations appear at the study site. That creates favourable conditions for the spread of local parasite lineages to migrating birds at stopover sites. Additionally, the local biting midges can acquire parasites with a more northerly origin during a blood meal on migrating birds and then transmit the infections to local birds. This could contribute to the geographical spread of haemosporidiosis agents [59]. Only *C. obsoletus* and *C. scoticus* were infected with haemosporidian parasites in autumn, so these insects are likely vectors during this season. Additional studies on the diversity and phenology of vector species are needed to improve understanding of the mechanisms underpinning the seasonal and spatial distribution of avian haemosporidian infections.

Application of commonly used nested PCR underestimates the number of co-infections with haemosporidians, as has been the case in many other studies [60]. The nested-multiplex PCR detected two *Haemoproteus/Plasmodium* co-infections in biting midges which were not detected by nested PCR thus is more preferable to use in parasite biodiversity studies [35].

Recently, there has been a significant increase in interest in the ecology of vectors as a major factor in the transmission of avian malaria parasites and other haemosporidians. These infections exhibit a global distribution; however, knowledge of specificity to vectors and patterns of disease epidemiology remains scarce. This is unfortunate because lack of such information precludes a better understanding of the detailed mechanisms of infection transmission, disease distribution and the development of prevention measures [13], which are particularly complicated in the case of migrating birds. Research on haemoproteid vectors is an alarming issue because some *Haemoproteus* parasites have been recently reported to cause disease and even lethal pathology in non-adapted avian hosts due to organ damage by exo-erythrocytic stages [61]. Furthermore, high parasitemia of these parasites is virulent in blood-sucking insects and can even cause their mortality [43, 55]. Further studies are needed for a better understanding of *Culicoides* species in the transmission of blood parasites.

## Conclusions

This study developed and tested a new easy-to-use methodology for the determination of natural vectors of avian haemosporidian parasites in the wild. Application of the PCR-based and microscopic tools in parallel is the essence of this methodology, which simplifies detection of sporozoites of haemosporidians in PCR-positive wild-caught blood-sucking insects. Experimental determination of vectors is difficult in wildlife studies, particularly in remote areas. Therefore, we recommend that this new approach is applied in vector and epidemiology studies in the wild. *Culicoides kibunensis* is a new vector species of avian haemoproteids and is a natural vector of *Haemoproteus pallidus*. Ornithophily of seven *Culicoides* species has been demonstrated, and species which are worth particular attention as possible active *Haemoproteus* parasite vectors are highlighted. Among these, *C. obsoletus*, *C. scoticus* and *C. kibunensis* are most important as possible natural vectors. This study contributes to the epidemiology of avian *Haemoproteus* infections by specifying *Culicoides* species that are likely responsible for the transmission of these pathogens in Europe.

## Additional files

**Additional file 1: Table S1.** The numbers of parous *Culicoides* biting midges of each species dissected weekly in Verkiai regional park, 2016. All collected parous females were dissected, except for those collected on 26th May, 2nd June and 22nd June, when the abundance of biting midges was high and only some of the sampled insects were dissected. Data on the species composition and numbers of dissected biting midges each week are provided.

**Additional file 2: Table S2.** Measurements of sporozoites detected in *Culicoides kibunensis* biting midges. Widths, lengths (μm) and areas of *Haemoproteus pallidus* sporozoites detected in *Culicoides kibunensis* biting midges are provided.

## Data Availability

Data supporting the conclusions of this article are included within the article and its additional files. The datasets used during the present study are available from the corresponding author. The newly generated sequences were submitted to the GenBank database under the accession numbers KM116567-KM116579. Voucher material was deposited at the Nature Research Centre, Vilnius, Lithuania under the accession numbers 49025-49026NS.

## Abbreviations

PCR: polymerase chain reaction
BLAST: Basic Local Alignment Search Tool
