# Supplementary material for: Identification of a new vector species of avian haemoproteids, with a description of methodology for the determination of natural vectors of haemosporidian parasites
Source: Parasit Vectors. 2019 Jun 18;12:307. doi: 10.1186/s13071-019-3559-8 (PMC6582567; doi:10.1186/s13071-019-3559-8)
Supplement: Supplementary file 1 — Additional file 1: Table S1. The numbers of parous Culicoides biting midges of each species dissected weekly in Verkiai regional park, 2016. All collected parous females were dissected, except for those collected on 26th May, 2nd June and 22nd June, when the abundance of biting midges was high and only some of the sampled insects were dissected. Data on the species composition and numbers of dissected biting midges each week are provided. [file 13071_2019_3559_MOESM1_ESM.doc]

**Additional file 1: Table S1.** The numbers of parous *Culicoides* biting midges of each species dissected weekly in Verkiai regional park, 2016. All collected parous females were dissected, except for the days 26^th^ May, 2^nd^ June and 22^nd^ June, when the abundance of biting midges was high and only part of sampled insects was dissected.

| Species |  | |  |  | | | | |  |  | | | |  | Month and day | | | | |  |  | | | |  |  | |
| --- | --- | --- | --- | --- | --- | --- | --- | --- | --- | --- | --- | --- | --- | --- | --- | --- | --- | --- | --- | --- | --- | --- | --- | --- | --- | --- | --- |
|  | May | |  | June | | | | |  | July | | | |  | August | | | | |  | September | | | |  | October | |
|  | 12 | 26 |  | 2 | 10 | 17 | 22 | 30 |  | 8 | 13 | 21 | 28 |  | 4 | 11 | 19 | 24 | 31 |  | 12 | 16 | 23 | 30 |  | 7 | 14 |
| *C. albicans* |  |  |  |  |  |  |  | 1 |  | 1 |  |  |  |  |  |  |  |  |  |  |  |  |  |  |  |  |  |
| *C. chiopterus* |  | 9 |  | 4 |  | 2 | 1 |  |  |  |  |  | 2 |  |  |  |  |  |  |  |  | 2 |  | 2 |  | 1 |  |
| *C. circumscriptus* |  |  |  |  | 3 | 1 |  |  |  |  |  |  |  |  |  | 1 |  |  |  |  |  |  |  |  |  |  |  |
| *C. fascipennis* |  | 2 |  |  |  |  |  |  |  |  |  |  |  |  |  |  |  |  |  |  |  |  |  |  |  |  |  |
| *C. festivipennis* |  |  |  |  | 4 | 3 | 7 | 3 |  | 2 |  | 1 |  |  |  |  |  |  |  |  |  |  |  |  |  |  |  |
| *C. impunctatus* |  |  |  |  | 12 | 14 | 4 | 6 |  |  |  |  |  |  |  |  |  |  |  |  |  |  |  |  |  |  |  |
| *C. kibunensis* |  |  |  |  |  | 12 | 38 | 14 |  | 3 | 1 |  |  |  |  |  |  |  |  |  |  |  |  |  |  |  |  |
| *C. newsteadi* |  |  |  |  |  |  |  | 1 |  |  |  |  |  |  |  |  |  |  |  |  |  |  |  |  |  |  |  |
| *C. obsoletus* |  | 16 |  | 36 | 5 | 4 |  |  |  | 4 | 4 |  | 7 |  |  |  | 4 | 4 |  |  | 6 | 12 |  | 15 |  | 6 |  |
| *C. pallidicornis* |  | 2 |  |  |  |  |  |  |  |  |  |  |  |  |  |  |  |  |  |  |  |  |  |  |  |  |  |
| *C. pictipennis* | 10 | 17 |  | 1 |  |  |  |  |  |  |  |  |  |  |  |  |  |  |  |  |  |  |  |  |  |  |  |
| *C. punctatus* |  | 8 |  | 12 | 8 | 3 | 1 | 4 |  |  |  | 6 | 8 |  | 1 | 2 | 3 | 3 | 2 |  | 4 | 26 |  |  |  |  |  |
| *C. reconditus* |  |  |  |  |  |  |  |  |  | 2 | 1 |  |  |  |  |  |  |  |  |  |  |  |  |  |  |  |  |
| *C. scoticus* |  | 1 |  |  |  |  |  |  |  | 1 | 1 |  | 4 |  |  |  |  | 4 | 4 |  | 8 | 14 |  | 9 |  | 17 |  |
| *C. segnis* |  |  |  |  |  |  |  | 4 |  | 7 | 4 | 1 | 1 |  |  |  |  |  |  |  |  |  |  |  |  |  |  |
